# Supplementary material for: MiR-182 Is Upregulated in Prostate Cancer and Contributes to Tumor Progression by Targeting MITF
Source: Int J Mol Sci. 2023 Jan 17;24(3):1824. doi: 10.3390/ijms24031824 (PMC9914973; doi:10.3390/ijms24031824)
Supplement: Supplementary file 1 [file ijms-24-01824-s001.zip › ijms-2093955-supplementary.pdf]

Table S1. Functional enrichment analysis of miR-182 related to TGF-β signaling

Table shows the significant association of miR-182 target genes with Gene Set descriptions related to TGF-β signaling. Analysis performed using clusterProfiler in CancerMIRNome.

Knowledge base	Gene Set ID	Description	Count / List Total	*p*-value	Adjusted *p*-value [1]	Gene Symbol
**TGF-β signaling**						
GO-BP	GO:0030511	Positive regulation of transforming growth factor beta receptor signaling pathway	4/179	1.15×10^{-4}	6.64×10^{-3}	EP300; SMAD4; THBS1; CITED2
	GO:1903846	Positive regulation of cellular response to transforming growth factor beta stimulus	4/179	1.15×10^{-4}	6.64×10^{-3}	EP300; SMAD4; THBS1; CITED2
	GO:0071560	Cellular response to transforming growth factor beta stimulus	9/179	3.64×10^{-4}	1.02×10^{-2}	EP300; CREB1; SMAD4; ACVR1B; THBS1; CITED2; NR3C1; ZFP36L1; LOX
	GO:0071559	Response to transforming growth factor beta	9/179	4.33×10^{-4}	1.17×10^{-2}	EP300; CREB1; SMAD4; ACVR1B; THBS1; CITED2; NR3C1; ZFP36L1; LOX
	GO:0007179	Transforming growth factor beta receptor signaling pathway	7/179	1.91×10^{-3}	3.18×10^{-2}	EP300; CREB1; SMAD4; ACVR1B; THBS1; CITED2; LOX

GO-BP = Gene Ontology-Biological Process. [1] Adjusted *p*-value for multiple hypothesis correction used Benjamini and Hochberg procedure.

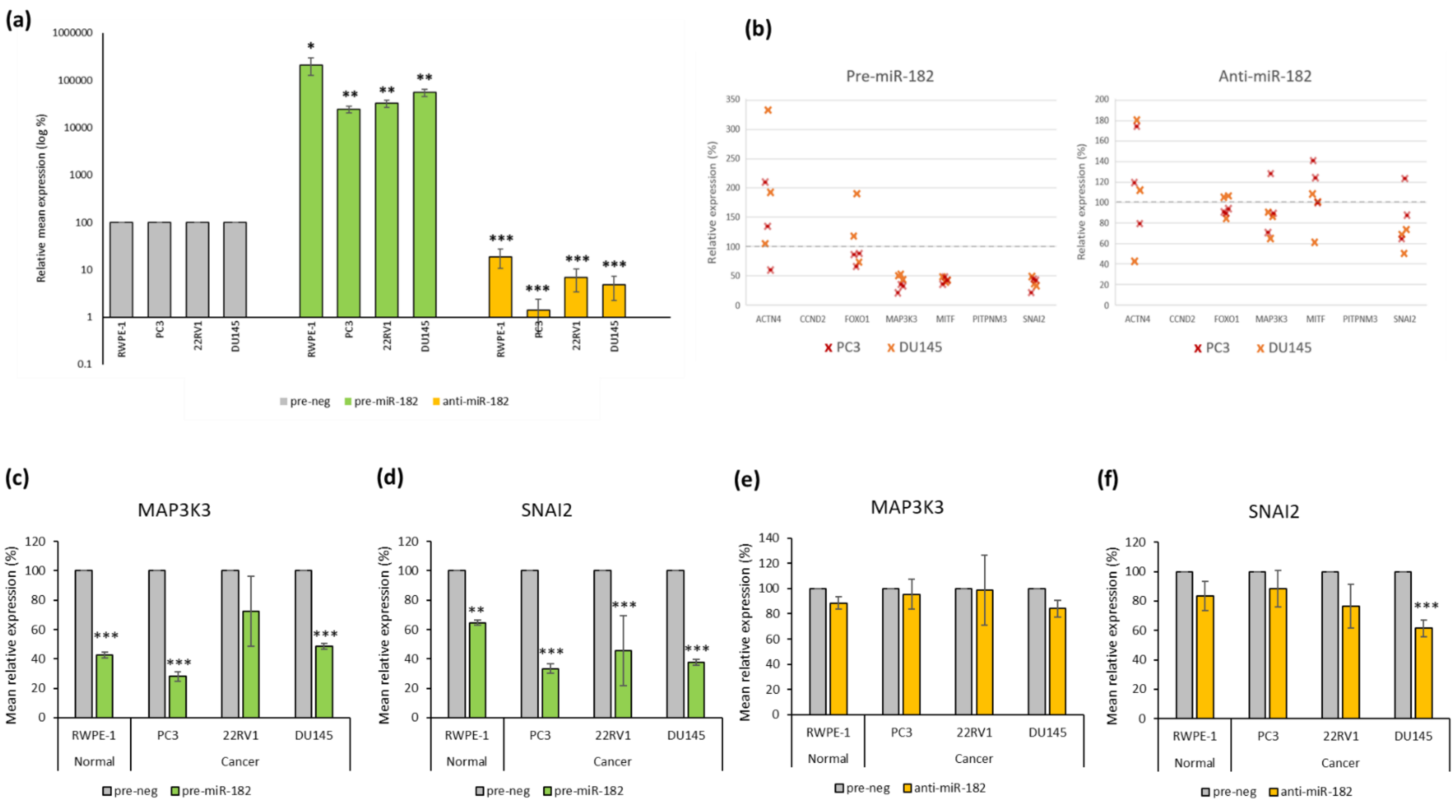

Figure S1. Effect of miR-182 expression on candidate targets. (a) RT-qPCR expression results of miR-182 48 hours post-transfection confirmed successful transfection of pre-miR-182 and anti-miR-182 in all cell-lines, where miR-182 was significantly upregulated (green) and downregulated (yellow) respectively, compared to control transfected cells ($n = 4$, pre-neg: negative control, housekeeping: Snord48). (b) In vitro preliminary RT-qPCR results of the 7 candidate targets. In pre-miR-182 transfected PC3 and DU145 cells, MAP3K3, MITF and SNAI2 expressions were consistently decreased, while ACTN4 and FOXO1 showed a wide range of expressions. In anti-miR-182 transfected cells, results were less consistent. CCND2 and PITPNM3 were undetectable in both cell lines ($n = 3$ per cell-line, each data point = one replicate vs pre-neg result normalised to 100%; housekeeping: ACTB). RT-qPCR shows over-expression of miR-182 causes significant down-regulation of (c) MAP3K3 and (d) SNAI2 in normal and cancerous prostate cell-lines ($n = 4$; housekeeping: ACTB). However, RT-qPCR ($n = 4$) shows inhibition of miR-182 does not cause up-regulation of (e) MAP3K3 and (f) SNAI2 in normal and cancerous prostate cell-lines ($n = 4$; housekeeping: ACTB). For all bar graphs, p-values generated by paired *t*-test (*$p < 0.05$, ** $p < 0.01$, *** $p < 0.001$).

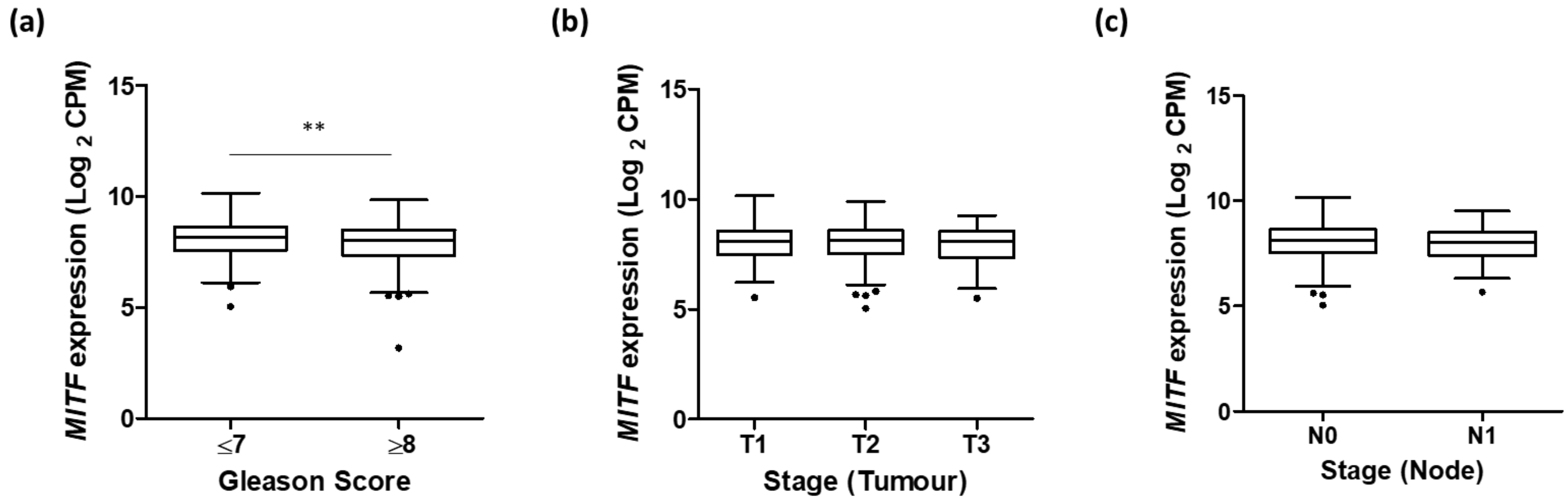

Figure S2. Correlation between MITF expression and clinicopathological markers of PCa progression

UCSC Xena analysis of TCGA PRAD samples shows expression of *MITF* is significantly lower in patients (a) with Gleason score ≥ 8 (n = 213) compared to those scored ≤ 7 (n = 337). There was no significant association between MITF expression and (b) pathological T-stage (n, T1 = 199, T2 = 190, T3 = 56)(One-way ANOVA with multiple comparison tests, p = ns) or (c) pathological N stage (n, N0 = 317, N1 = 66) (Welch's t-test, p = ns). All Boxplots show mean and Tukey whiskers. n, number; ns, non-significant.

Table S2. STRING functional enrichment analysis of MITF protein network using KEGG annotation. Table shows top 20 significantly enriched items ranked by Enrichment strength (ES)(Adjusted *p*-value < 0.05). Results are dominated by cancer-related annotations, including PCa (red), and EMT-related pathways (green) and function (purple).

ID	Description	Count in network	ES	Adjusted p-value [1]	Protein labels
hsa05219	Bladder cancer	4 of 41	1.96	1.83×10^{-6}	MAPK1, MDM2, MAP2K2, MAPK3
hsa05211	Renal cell carcinoma	6 of 66	1.93	4.97×10^{-9}	MAPK1, CREBBP, MAP2K2, MAPK3, EP300, TFE3
hsa05216	Thyroid cancer	3 of 36	1.89	6.92×10^{-5}	MAPK1, MAP2K2, MAPK3
hsa04720	Long-term potentiation	5 of 64	1.86	2.19×10^{-7}	MAPK1, CREBBP, MAP2K2, MAPK3, EP300
hsa05221	Acute myeloid leukaemia	5 of 66	1.85	2.36×10^{-7}	MAPK1, RPS6KB1, SPI1, MAP2K2, MAPK3
hsa04916	Melanogenesis	7 of 95	1.84	1.55×10^{-9}	MAPK1, CREBBP, MAP2K2, MAPK3, EP300, MITF, CREB1
hsa05215	**Prostate cancer**	7 of 96	1.83	1.55×10^{-9}	MAPK1, MDM2, CREBBP, MAP2K2, MAPK3, EP300, CREB1
hsa05218	Melanoma	5 of 72	1.81	3.15×10^{-7}	MAPK1, MDM2, MAP2K2, MAPK3, MITF
hsa04350	**TGF-β signaling pathway**	6 of 91	1.79	2.71×10^{-8}	MAPK1, RPS6KB1, SMAD2, CREBBP, MAPK3, EP300
hsa05210	Colorectal cancer	5 of 82	1.75	5.24×10^{-7}	MAPK1, RPS6KB1, SMAD2, MAP2K2, MAPK3
hsa04520	**Adherens junction**	4 of 67	1.75	8.95×10^{-6}	MAPK1, CREBBP, MAPK3, EP300
hsa04066	**HIF-1 signaling pathway**	6 of 106	1.72	5.19×10^{-8}	MAPK1, RPS6KB1, CREBBP, MAP2K2, MAPK3, EP300
hsa04068	**FoxO signaling pathway**	7 of 127	1.71	3.60×10^{-9}	SIRT1, MAPK1, MDM2, CREBBP, MAP2K2, MAPK3, EP300
hsa05214	Glioma	4 of 72	1.71	1.11×10^{-5}	MAPK1, MDM2, MAP2K2, MAPK3
hsa05212	Pancreatic cancer	4 of 73	1.71	1.14×10^{-5}	MAPK1, RPS6KB1, SMAD2, MAPK3
hsa05220	Chronic myeloid leukaemia	4 of 75	1.70	1.23×10^{-5}	MAPK1, MDM2, MAP2K2, MAPK3
hsa04960	Aldosterone-regulated sodium reabsorption	2 of 37	1.70	0.0028	MAPK1, MAPK3
hsa01522	Endocrine resistance	5 of 95	1.69	8.74×10^{-7}	MAPK1, RPS6KB1, MDM2, MAP2K2, MAPK3
hsa04370	**VEGF signaling pathway**	3 of 57	1.69	0.00021	MAPK1, MAP2K2, MAPK3
hsa05213	Endometrial cancer	3 of 57	1.69	0.00021	MAPK1, MAP2K2, MAPK3

[1]Adjusted *p*-value for multiple hypothesis correction used Benjamini and Hochberg procedure.

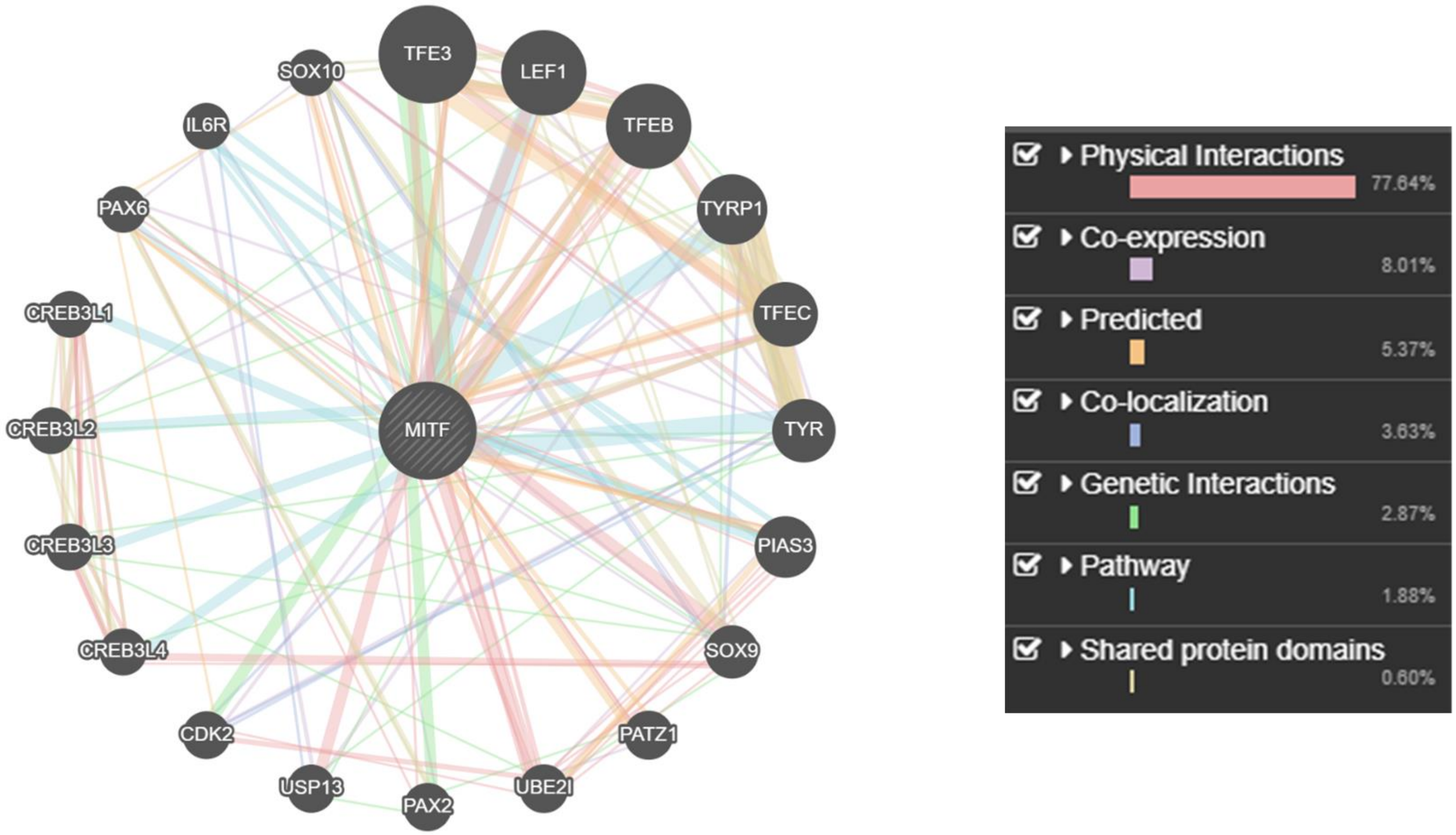

Figure S3. Network analysis of MITF interactions. Visualization by GeneMANIA.

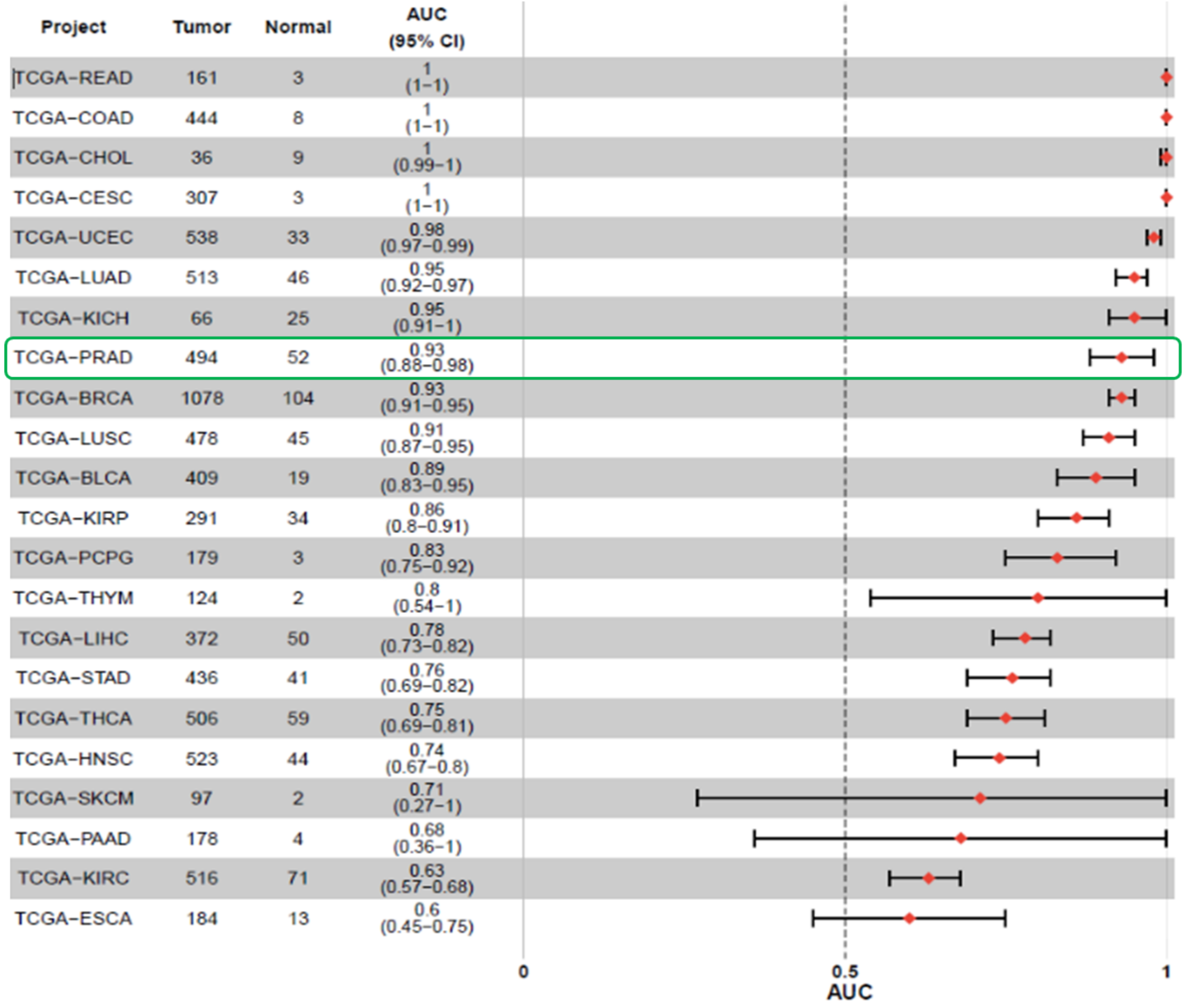

Figure S4. CancerMIRNome TCGA Pan-Cancer ranked forest plots of miR-182-5p ROC analysis revealed in general that miR-182-5p has significant diagnostic value in multiple cancer types (AUC = 0.6 - 1.0). Result for PCa is enclosed in green circle (TCGA-PRAD).

AUC: Area under the curve; OS: Overall survival; ROC: Receiver operating characteristic; BLCA: Bladder urothelial carcinoma; BRCA: Breast invasive carcinoma; CESC: Cervical squamous cell carcinoma and endocervical adenocarcinoma; CHOL: Cholangiocarcinoma; COAD: Colon adenocarcinoma; ESCA: Esophageal carcinoma; HNSC: Head and neck squamous cell carcinoma; KICH: Kidney chromophobe; KIRC: Kidney renal clear cell carcinoma; KIRP: Kidney renal papillary cell carcinoma; LIHC: Liver hepatocellular carcinoma; LUAD: Lung adenocarcinoma; LUSC: Lung squamous cell carcinoma; PAAD: Pancreatic adenocarcinoma; PCPG: Pheochromocytoma and paraganglioma; PRAD: Prostate adenocarcinoma; READ: Rectal adenocarcinoma; SKCM: Skin cutaneous melanoma; STAD: Stomach adenocarcinoma; THCA: Thyroid carcinoma; THYM: Thymoma; UCEC: Uterine corpus endometrial carcinoma

Table S3 KM plotter meta-analysis results of overall survival in various cancers comparing high/low expression of miR-182 and MITF. Table included results with log-rank $p < 0.05$. HR estimated for high compared to low expression (Cox proportional analysis, auto-selected cut-off).

	Cancer	*n*	*HR**	*Log-rank p-value*
miR-182	Breast cancer	1076	0.70	0.035
	Head-neck squamous cell carcinoma	522	0.70	0.013
	Kidney renal clear cell carcinoma	516	1.94	2.2×10^{-5}
	Kidney renal papillary cell carcinoma	290	0.52	0.037
	Liver hepatocellular carcinoma	371	1.56	0.012
	Lung squamous cell carcinoma	472	0.57	0.00016
	Sarcoma	259	1.78	0.0043
	Stomach adenocarcinoma	431	0.68	0.012
	Uterine corpus endometrial carcinoma	537	0.51	0.0021
MITF	Cervical squamous cell carcinoma	304	0.62	0.043
	Esophageal adenocarcinoma	80	0.37	0.0028
	Kidney renal clear cell carcinoma	530	0.55	8.3×10^{-5}
	Kidney renal papillary cell carcinoma	287	0.51	0.031
	Liver hepatocellular carcinoma	370	1.54	0.016
	Lung adenocarcinoma	504	0.66	0.019
	Ovarian cancer	373	1.47	0.0054
	Pancreatic ductal adenocarcinoma	177	2.09	0.0039
	Rectum adenocarcinoma	165	2.24	0.037
	Sarcoma	259	0.54	0.0019
	Thymoma	118	3.96	0.027

* Red text indicates HR < 1; high expression of miR-182/MITF predicted favourable prognosis; blue text indicates HR > 1; high expression of miR-182/MITF predicted poor prognosis. *n* = number; HR = Hazard ratio; KM = Kaplan-Meier

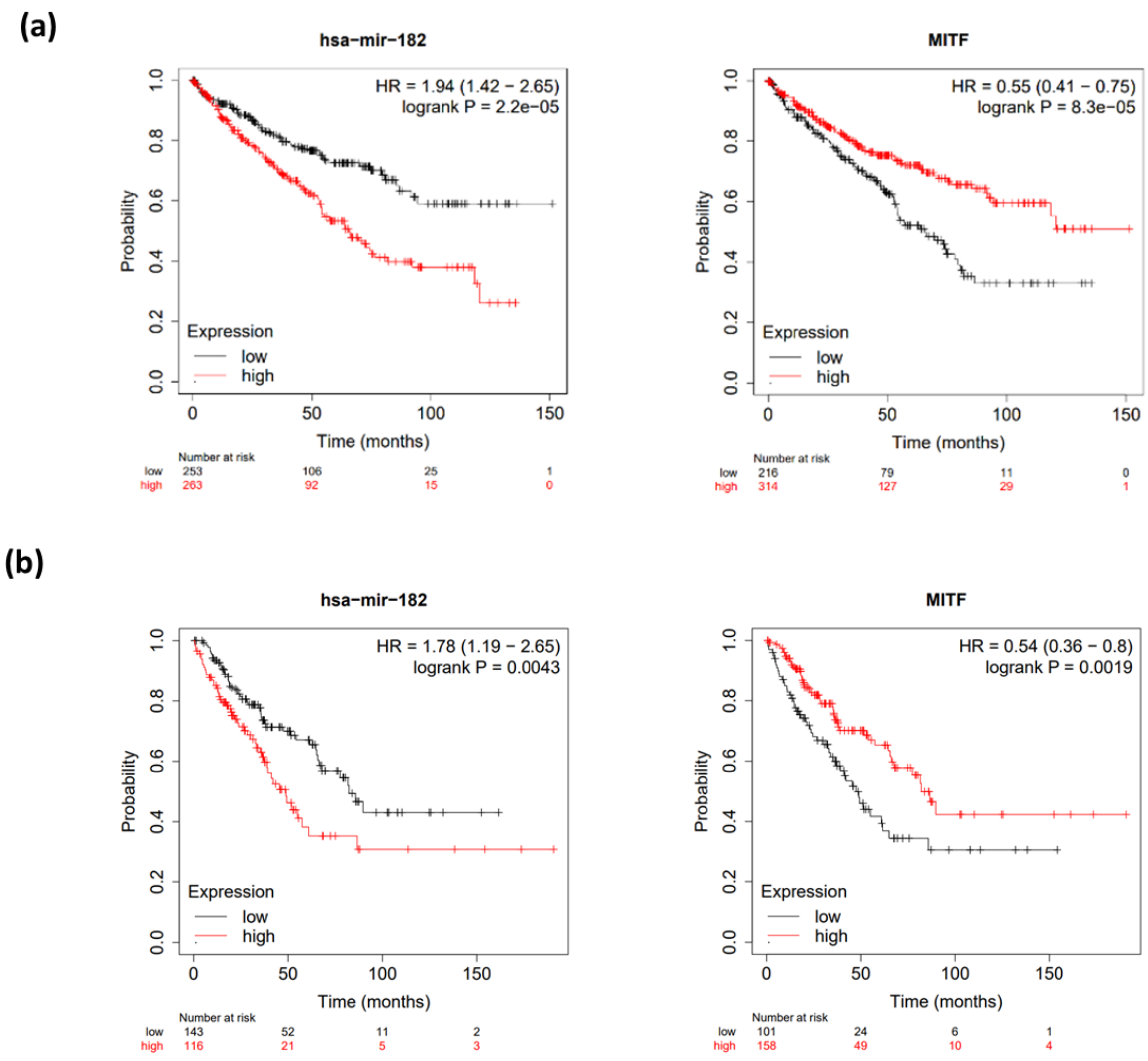

Figure S5. KM survival plots showing significant inverse associations between miR-182 and MITF expressions in **(a)** Kidney renal clear cell carcinoma (*n*: miR-182 = 516, MITF = 530), and **(b)** Sarcoma (*n*: miR-182 = 259, MITF = 259). In both cases, high miR-182 expression is significantly associated with poor prognosis, while high MITF is significantly associated with favourable prognosis. Visualization by KM Plotter. *n* = number; KM = Kaplan-Meier

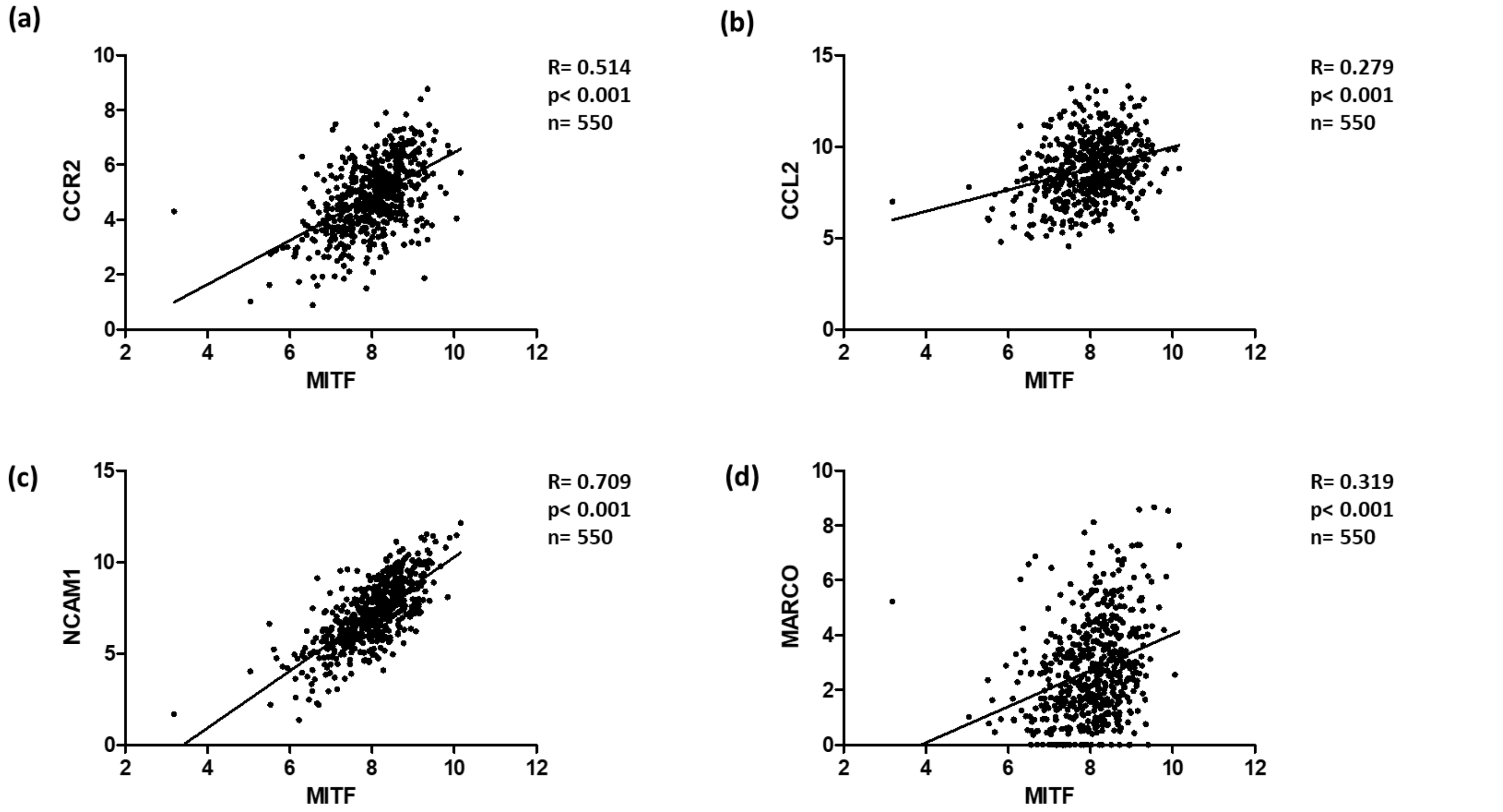

Figure S6. Correlation of MITF with gene markers of tumor-associated macrophages. UCSC Xena analysis of TCGA prostate tissue samples (*n* = 550) , shows the expression of MITF is significantly positively correlated with (a) CCR2 (b) CCL2 (c) NCAM1 (d) MARCO (Pearson correlation, $p < 0.001$). *n* = number

Table S4. Diagnostic and prognostic studies of miR-182-5p in prostate cancer.

miR-182-5p	Source	Diagnostic	Prognostic		Reference
Up	Plasma	65 PCa vs 58 BPH<br>AUC = 0.61<br>(*p* = 0.0365)	(NA)		*[59]*<br>*Abramovic et al., 2021*
Up	Tissue	82 PCa vs 24 BPH<br>AUC = 0.95<br>(*p* < 0.001)	**BCR-free survival**<br>34 AA PCa:<br>HR = 2.93<br>(*p* = 0.041)	 22 EA PCa:<br>HR = 0.83<br>(*p* = 0.86)	*[60]*<br>*Shiina et al., 2021*
Up	Tissue	98 PCa vs 15 Normal<br>AUC=0.81<br>(*p* = 0.0001)	(NA)		*[61]*<br>*Bidarra et al., 2019*
	Plasma	252 PCa vs 52 Normal<br>AUC = 0.64<br>(*p* = 0.0021)	**MFS** (252 PCa)<br>Univariate:<br>Log-rank *p* = 0.0206	 Multivariate:<br>(Not significant)	
Up	Urine<br>Tissue	(Urine)<br>47 PCa vs 45 Normal<br>OR = 0.976<br>(*p* = 0.717)	(Tissue, 272 PCa)<br>**Biochemical PFS**<br>Log-rank *p* = 0.026<br>**Clinical PFS**<br>Log-rank *p* = 0.043		*[62]*<br>*Casanova-Salas et al., 2014*
Up	Tissue	(NA)	**BCR-free survival** (100 PCa)<br>Univariate OR = 1.91 (*p* = 0.11)<br>**MFS** (74 PCa)<br>Univariate OR = 1.07 (*p* = 0.93)		*[63]*<br>*Wallis et al., 2015*

AA = African Americans; AUC = Area under the curve; BCR = Biochemical recurrence; BPH = Benign prostatic hyperplasia; EA = European Americans; MFS = Metastatic-free survival; NA = Not applicable; OR = Odds ratio; PFS = Progression-free survival
